# Tobacco cessation intervention for pregnant women in Argentina and Uruguay: study protocol

**DOI:** 10.1186/1742-4755-10-44

**Published:** 2013-08-26

**Authors:** Fernando Althabe, Alicia Alemán, Agustina Mazzoni, Mabel Berrueta, Paola Morello, Mercedes Colomar, Alvaro Ciganda, Ana Becú, Luz Gibbons, Laura Llambi, María G Bittar Gonzalez, Van T Tong, Sherry L Farr, Ruben A Smith, Patricia M Dietz, Carolyn Johnson, Pierre Buekens, José M Belizán

**Affiliations:** 1 Institute for Clinical Effectiveness and Health Policy (IECS), Dr. Emilio Ravignani 2024, C1414CPV, Buenos Aires, Argentina; 2 Montevideo Clinical and Epidemiological Research Unit (UNICEM), Montevideo, Uruguay; 3 Clínica Médica "A" & Tobacco Cessation Clinic, Hospital de Clínicas, Facultad de Medicina, Universidad de la República, Montevideo, Uruguay; 4 Preventive Medicine Department, Facultad de Medicina, Universidad de la República, Montevideo, Uruguay; 5 Division of Reproductive Health, National Center for Chronic Disease Prevention and Health Promotion, Centers for Disease Control and Prevention (CDC), Atlanta, GA, USA; 6 Tulane School of Public Health and Tropical Medicine, 1440 Canal Street, Suite 2301 School of Public Health, New Orleans, LA, 70112, USA

**Keywords:** Pregnancy, Smoking cessation, Guidelines

## Abstract

**Background:**

Argentina and Uruguay are among the countries with the highest proportion of pregnant women who smoke. The implementation of an effective smoking cessation intervention would have a significant impact on the health of mothers and infants. The “5 A’s” (Ask, Advise, Assess, Assist, Arrange) is a strategy consisting of a brief cessation counseling session of 5–15 minutes delivered by a trained provider. The “5 A’s” is considered the standard of care worldwide; however, it is under used in Argentina and Uruguay.

**Methods:**

We will conduct a two-arm, parallel cluster randomized controlled trial of an implementation intervention in 20 prenatal care settings in Argentina and Uruguay. Prenatal care settings will be randomly allocated to either an intervention or a control group after a baseline data collection period. Midwives’ facilitators in the 10 intervention prenatal clinics (clusters) will be identified and trained to deliver the “5 A’s” to pregnant women and will then disseminate and implement the program. The 10 clusters in the control group will continue with their standard in-service activities. The intervention will be tailored by formative research to be readily applicable to local prenatal care services at maternity hospitals and acceptable to local pregnant women and health providers. Our primary hypothesis is that the intervention is feasible in prenatal clinics in Argentina and Uruguay and will increase the frequency of women receiving tobacco use cessation counseling during pregnancy in the intervention clinics compared to the control clinics. Our secondary hypotheses are that the intervention will decrease the frequency of women who smoke by the end of pregnancy, and that the intervention will increase the attitudes and readiness of midwives towards providing counseling to women in the intervention clinics compared to the control clinics.

**Trial registration:**

ClinicalTrials.gov. Identifier: NCT01852617

## Background

Tobacco use in low- and middle-income countries (LMIC) has been increasing since 1970, while it has been stable or decreasing in high-income countries. It is estimated that tobacco use currently causes 5 million deaths per year worldwide and that it will cause 10 million deaths by the year 2020 [1]. Historically, the prevalence of smoking among women in the developing world has been very low, mostly because of strong cultural constraints against women smoking; approximately 50% of men in developing nations smoke cigarettes, compared with 9% of women [2].

### Tobacco epidemic in women in Argentina and Uruguay

Argentina and Uruguay are among the countries with the highest proportion of women who smoke, showing rates of 26% to 33% in different ages and subgroups [3]. Argentina has one of the highest smoking rates in the Americas. Reports from the National Risk Factors Survey (ENFR) conducted in 2005 [4] show that 28.6% of women ages 18–65 were current smokers and 13.4% were former smokers. More recent data from the 2008 National Survey of Use of Psychoactive Substances [5] show that 30.1% of women aged 18–64 were current smokers. Among adults, female smoking rates are still lower than male smoking rates. [4] In Uruguay, the smoking prevalence among women has steadily increased since 1990. Between 1993 and 1998, surveys have shown that between 17.4% and 27% of women of different age groups and locations were current smokers [6]. The Permanent National Survey of Households in Uruguay, which is performed annually in a random sample of households in the whole country, and included a question on tobacco use since 2006, showed that 29% of women between 20–29 years old were current smokers in that year [7]. The National Survey of Risk Factors conducted in 2006, with a sample of 2,010 men and women ages 25–64, showed that 32.7% of the population were current smokers. Among all women, 28.6% reported being current tobacco users, but this proportion increased to 33% in the age group of 25–44 years old [8]. All of the surveys also showed that smoking prevalence was still higher in men (32% to 38% in different groups and years) but that the difference with women is decreasing over time. Both Argentina and Uruguay, like other developing countries, seem to be moving into the third stage of the tobacco-use epidemic, with a growing prevalence of female smokers who will eventually exceed the prevalence in men.

### Tobacco use during pregnancy in Argentina and Uruguay

Pregnant women are a priority population for tobacco control efforts because cigarette smoking during pregnancy poses serious risks to fetal health. Smoking during pregnancy may cause preterm delivery, low birth weight, and sudden infant death syndrome [2,9]. Maternal tobacco use is also likely to expose infants and children to secondhand smoke (SHS) and to provide a role model for children’s use of tobacco [3].

There is scarce information on the prevalence of tobacco use during pregnancy in LMIC. Our group conducted the only published survey in Argentina and Uruguay designed to study pregnant women in prenatal clinics, which was part of a global survey performed in nine LMIC [2]. The survey, conducted during 2005 and 2006, showed that tobacco use during pregnancy is culturally acceptable in these countries and found rates of smoking during pregnancy of 10.3% in Argentina and 18.3% in Uruguay. The observed rates were lower for other Latin American countries like Brazil (6.1%), Ecuador (0.8%), and Guatemala (0.8%).

Reports from routinely collected data in a perinatal network of 30 public maternity hospitals attending approximately 90,000 deliveries per year in the Province and City of Buenos Aires in 2008 showed 15% of women reporting smoking at their first prenatal visit [10]. Similar reports from the largest public maternity hospital in Uruguay (Hospital Pereira-Rossell), attending 9,000 childbirths per year (15% of total births in the country), showed that the prevalence of women smoking more than 5 cigarettes per day during pregnancy was 19.5% in 2007 (Internal Report, Informatic Perinatal System, personal communication, C. Sosa).

Magri et al. reported a 33% prevalence of smoking during all pregnancies in a survey performed of 900 women after delivering in Hospital Pereira-Rossell [11]. Cotinine in meconium was tested in a sample of 10% of newborns, and positive determinations reached 51% [11].

The National Observatory of Gender and Reproductive and Sexual Health 2008 conducted a survey during postpartum stays in a sample of 564 women who underwent childbirth in public and private health institutions in Uruguay. Prevalence of smoking during pregnancy was 22% in this sample of women, and 77% reported not receiving systematic counseling interventions from their providers [12].

These studies suggest that Argentina and Uruguay are among the countries with the highest smoking rates during pregnancy in Latin America and probably worldwide.

### Evidence-based interventions for smoking cessation during pregnancy: the “5 A’s” strategy

A strong body of research supports that clinicians should consistently identify tobacco users and provide tobacco dependence counseling to patients who use tobacco. Brief cessation counseling interventions based on the “5 A’s” model (Ask, Advise, Assess, Assist, Arrange) have been proven effective to increase smoking cessation in a wide variety of settings, populations, and by all types of providers. The “5 A’s” strategy is currently considered the standard of care in the United States of America for all patients contacting clinicians, including pregnant women during prenatal care [13].

Specifically in pregnancy, randomized-controlled trials of brief cessation strategies based on the “5 A’s” have been summarized in several systematic reviews [13,14]. The reviews included 8 [13] to 16 studies [14] and reported a summary risk ratio of 1.7-1.8, with 95% confidence intervals (CI) of 1.3 to 2.2. These findings suggest a 70%-80% improvement in cessation rates in women receiving brief counseling, with a 95% confidence that there is at least a 30% improvement in cessation. The Cochrane review [15], which classified the interventions in a different way, found that women receiving cognitive behavior interventions showed a 5% (95%CI 3%-8%) absolute reduction in the number who continued smoking. The intervention has been proven effective in different ethnic groups (African Americans and non-Hispanic whites) and has been delivered by different types of trained health providers, including physicians, midwives, and nurses. It is also effective among individuals with low socioeconomic status or limited formal education (odds ratio [OR] = 1.42; 95%CI 1.04–1.92) [13]. The “5 A’s” intervention currently recommended for pregnant women is summarized in Table 1[14].

**Table 1:** “5 A’s” Intervention

**Step 1: ASK**	Ask the patient about her smoking status:
	A. I have **NEVER** smoked, or I have smoked less than 100 cigarettes in my lifetime.
	B. I stopped smoking **BEFORE** I found out I was pregnant, and I am not smoking now.
	C. I stopped smoking **AFTER** I found out I was pregnant, and I am not smoking now.
	D. I smoke some now, but I cut down on the number of cigarettes I smoke **SINCE** I found out I was pregnant.
	E. I smoke regularly now, about the same as **BEFORE** I found out I was pregnant.
**Step 2: ADVISE**	Provide clear, strong advice to quit with personalized messages about the impact of smoking and quitting on the mother and fetus.
**Step 3: ASSESS**	Assess the willingness of the patient to make a quit attempt within the next 30 days.
**Step 4: ASSIST**	- Provide pregnancy-specific, self-help smoking cessation materials.
	- Suggest and encourage the use of problem solving methods and skills for cessation.
	- Arrange social support in the smoker’s environment.
	- Provide social support as part of the treatment.
**Step 5: ARRANGE**	- Periodically assess smoking status and, if she is a continuing smoker, encourage cessation.

The evidence for brief cessation counseling in pregnancy is based on studies conducted in high-income countries, mainly the U.S. but also Australia and Sweden. Although no trials were conducted in Argentina and Uruguay, there are no serious reasons to expect that the observed effect of the “5 A’s” intervention would not be similar in these two countries as: 1) a large proportion of the population of women in Argentina and Uruguay are from European origin (Italian and Spanish immigrants) [4,16], 2) more than 95% of women completed elementary school [4,16], 3) 99% of deliveries are attended at hospitals; more than 90% of pregnant women receive prenatal care, and 4) similar kinds of prenatal care providers are involved (physicians or midwives).

### Tobacco control policies in Argentina and Uruguay

Strategies to promote smoking cessation in the general population are a key component of the national tobacco control program of the Ministry of Health in Argentina. In 2004, a panel of experts reviewed the existing data on smoking cessation and developed a national clinical practice guideline [17]. The recommendations included in the clinical guidelines are very similar to the current recommendations by the U.S. Department of Health and Human Services in 2008 [13], which recommend that providers identify all tobacco users and provide appropriate interventions if patient is willing to quit, based on the “5 A’s” model intervention.

In Uruguay, the Ministry of Health created the National Program for Tobacco Control in 2005 with aims to decrease the prevalence of tobacco use among the population by decreasing the number of new users and by promoting smoking cessation among current users. The program also promotes the implementation of strategies to ensure smoke-free areas in the whole country. Since 2005, the National Fund of Resources, a governmental agency, and the National Commission on the Fight against Cancer (an independent fund), have been implementing workshops all over the country to train health care providers in smoking cessation skills [18]. The National Guidelines for Tobacco Management in Uruguay include a specific chapter for smoking cessation among pregnant women and recommend that clinicians provide brief counseling as the main strategy for cessation among pregnant women [19].

### Use of the “5 A’s” intervention in pregnant women in Argentina and Uruguay

Despite national recommendations and dissemination efforts, pregnant women attending prenatal care at public maternity hospitals and prenatal clinics are not routinely receiving brief counseling. In 2010, we surveyed 16 public maternity hospitals participating in a perinatal network and four hospitals in Uruguay. Directors and coordinators of the obstetric departments were asked if the hospital offered smoking cessation programs on a routine basis to pregnant women, what proportion of women attending the hospital’s prenatal care clinics were screened for tobacco use during each prenatal visit, and what proportion of women identified as current smokers received brief cessation counseling or were referred for specialized advice. Of the 16 hospitals, only 6 (37%) responded that they had specific guidelines or recommendations. The median proportion of women screened for tobacco use in every prenatal visit was 5% (range 0–100, 8 hospitals reported 0% and 3 reported 100%), and only 10% of pregnant smokers were receiving brief counseling.

Based on a 2005 survey of obstetrician/gynecologists in Argentina, only 22% had received training in smoking cessation counseling and 48.5% reported insufficient knowledge to provide smoking cessation advice. Although 88.9% always or almost always advised women to stop smoking, 75% believed it was acceptable for pregnant women to smoke up to 6 cigarettes per day [20].

### Barriers to the adoption of evidence-based practices for tobacco cessation during pregnancy

A wide variety of barriers can hinder practitioners from adhering to evidence-based practices. Barriers to the implementation of clinical guidelines can be classified by factors related to health providers (knowledge, attitudes and behavior), patients, and the environment [21,22].

Regarding factors affecting the use of smoking cessation interventions in antenatal clinics, one study conducted in Australia investigated perceptions, knowledge, and the use of brief interventions in midwives and physicians working in 20 hospital antenatal clinics [23]. Results showed that the majority of antenatal clinic staff did not use the most effective forms of brief cessation interventions. Additionally, results showed that the presence of specific procedures and training in smoking cessation intervention appeared to be the most important predictors of smoking intervention use in those clinics.

A recently published systematic review of 23 studies identified 10 aspects of service delivery relating to the uptake of interventions for smoking cessation among pregnant women. These were whether or not the subject of smoking is broached by a health professional, the content of advice and information provided, the manner of communication, having service protocols, follow-up discussion, staff confidence in their skills, the impact of time and resource constraints, staff perceptions of ineffectiveness, differences between professionals, and obstacles to accessing interventions [24].

### Strategies to disseminate and implement smoking cessation programs and clinical practice guidelines in maternal and child health services

We have identified two trials that evaluated interventions to disseminate smoking cessation programs at antenatal clinics [25,26]. Both studies evaluated interventions to actively disseminate brief intervention strategies for smoking cessation that were recommended but not used in antenatal clinics.

Cooke and colleagues conducted a cluster-randomized trial in 23 hospital clinics in Australia. Clinics were randomly allocated to two groups, which received the program of brief intervention for smoking cessation either by simple or intensive dissemination methods. Simple dissemination involved materials sent by mail, and intensive dissemination added personal contact with trained midwives acting as facilitators who provided support and training for the program [25]. The outcome assessment method was a telephone interview with a manager three months after the beginning of the program. Clinic managers were surveyed to assess their perceptions about the program and current use. The results showed that there were no differences in the adoption of the program in intervention and control clinics. Unfortunately, the outcome assessment method used in this trial was very likely to be subject to information bias and did not assess whether pregnant women actually received the brief intervention for smoking cessation.

Lowe and colleagues performed a cluster-randomized trial to evaluate the impact of a behaviorally-based intervention designed to increase the number of hospitals that routinely provide effective smoking cessation programs (brief intervention) for pregnant women in 70 public hospitals in Queensland, Australia [26]. Hospitals were randomly allocated to either a group receiving the program in printed materials, or to a group receiving training workshops and an implementation component including reminders were added. The main outcome was the rate of clinics implementing the smoking cessation program in more than 80% of the women, assessed by telephone interviews to the clinic medical director and nursing director. At one year of follow-up, the results showed that 15 (68%) of the intervention hospitals were providing antenatal smoking cessation to smoking pregnant women, compared with only 3 (14%) in the control hospitals. While the intervention was well-designed, the outcome assessment method was likely to be susceptible to information bias, and the actual proportion of women receiving the program intervention was not measured.

We have not identified dissemination trials of smoking cessation programs for pregnant women in Latin American countries or in LMIC. Trials of interventions to disseminate and implement clinical practice guidelines in maternal and child health in LMIC are scarce [27], and only one has been done in Latin America, conducted by our group. We conducted a multicenter, international cluster randomized trial in 19 public maternity hospitals in Argentina and Uruguay [28], evaluating a behavioral intervention to increase the use of evidence-based childbirth practices.

### Study hypotheses and aims

Our ***primary hypothesis*** is that an intervention designed to motivate, persuade, and train midwives to deliver the “5 A’s” strategy during antenatal care is feasible in public prenatal care settings in Argentina and Uruguay and will increase the frequency of women receiving tobacco use cessation counseling during pregnancy in the intervention clinics, compared to the standard in-service training practices at the control clinics. Our ***secondary hypotheses*** are that the intervention will decrease the frequency of women who smoke by the end of pregnancy, and that the intervention will increase the attitudes and readiness of midwives towards providing counseling to women in the intervention clinics compared to the control clinics.

The ***main specific aim*** of this project is to evaluate an intervention intended to increase the frequency of women that receive the “5 A’s” strategy during pregnancy. In an implementation cluster randomized-controlled trial, we will randomize 20 prenatal care settings in the Province of Buenos Aires, Argentina, and in Montevideo, Uruguay. Midwife facilitators in the 10 intervention prenatal care settings will be identified and trained on delivery of the “5 A’s” to pregnant women (training of the trainers) and on organizing the training and delivery at their facilities in order to reach all women in prenatal care. They will then disseminate and implement the program in their prenatal clinics. The intervention will be tailored by formative research to be readily applicable to local prenatal care services and to be acceptable to local pregnant women and health providers. Pregnancy-specific self-help materials will be adapted and their acceptability evaluated. The 10 prenatal care settings in the control group will continue with their standard in-service activities.

### Rationale for the trial

We have shown that a multifaceted intervention to implement clinical guidelines and programs is effective to increase the use of childbirth practices in public maternity hospitals in Argentina and Uruguay [28]. An intervention following a similar basis, which includes proven, effective strategies to disseminate guidelines or programs (interactive workshops, training on the “5 A’s,” use of reminders), is likely to succeed in implementing the program. Furthermore, a similar intervention was evaluated in Australia 10 years ago [25] and proved effective in increasing the smoking cessation program implementation in prenatal clinics, as reported by clinic managers.

## Methods

### Overview

**Figure 1:**
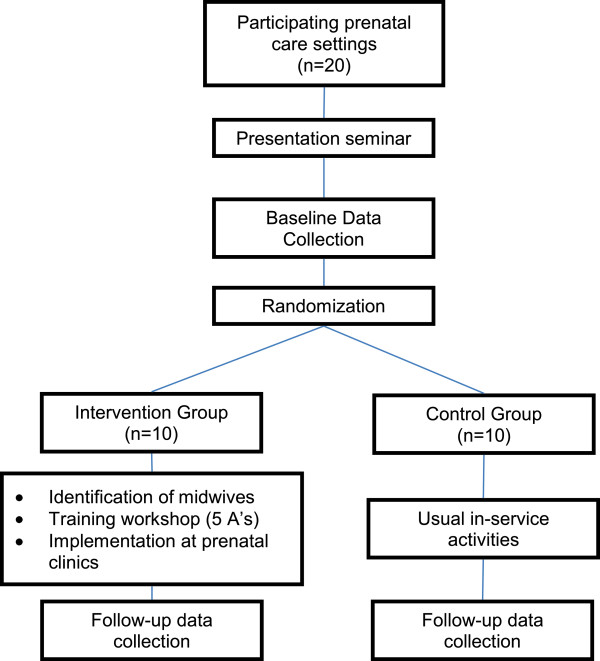
Trial design.

We will conduct a two-arm, parallel cluster randomized-controlled trial with baseline and follow-up cross sectional measurements in 20 prenatal care settings in Argentina and Uruguay. Prenatal care settings will be randomly allocated to either an intervention or a control group after a baseline data collection period. The intervention will include identification and training of midwife facilitators on the “5 A’s.” The facilitators will then disseminate and implement the smoking cessation program at their prenatal care settings. The prenatal care settings in the control group will continue with their standard in-service activities. A follow-up data collection period will be conducted in all hospitals. The intervention will be tailored by formative research to be readily applicable to local prenatal care services at busy maternity hospitals and to be acceptable to local pregnant women and health providers (Figure 1).

### Participating clusters

Argentina and Uruguay are middle-income countries with a highly literate population (97% of the population >15 years). Approximately, 99% of the childbirths are attended at maternity hospitals; 70% and 50% of childbirths take place in publicly-funded hospitals in Argentina and Uruguay, respectively, which serve the most underprivileged population. Prenatal care is provided by physicians and midwives, and over 94% of pregnant women receive prenatal care during at least four visits during pregnancy (a mean of 7 visits) [29,30].

In the public health sector, prenatal care is offered at prenatal clinics within the maternity hospitals or at primary health centers associated with them. At the hospitals, prenatal care providers are mostly obstetricians or obstetrical residents assisted by midwives; while at the primary health center, both midwives and obstetricians provide prenatal care. Our research is intended to benefit the most underprivileged populations in our region; as such, this study will be conducted in a sample of prenatal clinics within public maternity hospitals in Argentina and Uruguay and/or at primary health centers. We will call these sites “prenatal clinics,” irrespective of whether they are located at a hospital or a primary health center.

We invited public prenatal clinics located at maternity hospitals or primary health centers in the metropolitan area of the Province of Buenos Aires, Argentina, and in Montevideo, Uruguay, to participate in the study. All of them have agreed to participate in this project. The eligibility criteria for these prenatal clinics include: 1) not having a smoking cessation program based on the “5 A’s” for pregnant women in place, 2) having an estimated frequency of women receiving the “5 A’s” intervention below 20%, and 3) having midwives or nurse-midwives as part of the hospital or primary health center staff.

We will collect baseline data from those pre-selected prenatal clinics. According to the results of the analysis of the baseline data collection, prenatal clinics will be excluded if the frequency of women receiving the “5 A’s” intervention is 20% or higher. Because these prenatal clinics do not have smoking cessation program in place, it is expected that none of them will be excluded by this criterion. However, the sample size of the study was increased to allow for exclusions.

**Figure 2:**
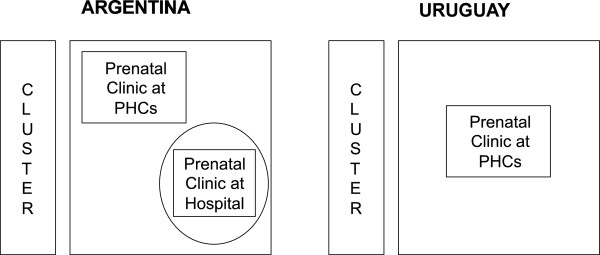
**Conformation of the cluster per country.** PHCs: Primary Health Centers.

In Argentina, prenatal care is organized in two different settings: until 34–36 weeks of gestational age, women attend their prenatal care at prenatal clinics located in primary health centers; after 34–36 weeks, they are referred by the health center’s provider to continue prenatal care in the maternity hospital’s prenatal clinic where they plan to deliver. Deliveries are attended in the maternity hospitals. In Uruguay, all prenatal care visits take place at the prenatal clinics located in primary health centers, and women only go to the maternity hospitals at the time of delivery. This different organization has implications in the conformation of the clusters in each country. In Argentina, the cluster will include the prenatal clinics of the primary health centers and the prenatal clinics of the maternity hospitals, and in Uruguay, each prenatal clinic of a primary health center will be defined as a cluster (Figure 2).

In Argentina, we have selected 10 clusters composed of the maternity hospitals and the associated prenatal clinics at health centers. Each hospital has two or three associated prenatal clinics, located in primary health centers in the surrounding area (where women attend prenatal care until 34–36 weeks of pregnancy). In Uruguay, we have selected 10 prenatal clinics; each clinic forms one cluster. All women receiving antenatal care at the participating prenatal clinics will be eligible to receive the “5 A’s” intervention.

### Randomization procedures

The conformation of the cluster will be different in each country. Thus, we will consider the following as a cluster: groups of prenatal clinics, composed by the prenatal clinic located inside the maternity hospital and the associated prenatal clinics located in the primary health centers of the area. Deliveries are assisted at the hospitals in both countries. Thus, the outcome will be assessed at the hospitals.

For cluster randomized trials with repeated cross-sectional binary measurements and a limited number of clusters, it is important to attempt to achieve as much balance as possible between the two arms of the study [31]. Based on the results of the initial assessment of health facility characteristics in the start-up phase of the study and using the baseline data, a “balanced randomization” technique [32] will be used to better achieve balance between the treatment groups in terms of the following characteristics of the prenatal clinics: frequency of women receiving the “5 A’s,” frequency of women who smoke during pregnancy, number of health providers/annual number of deliveries, proportion of midwives or nurse midwives who attend prenatal care/total of providers, and country. We have used similar randomization procedures in our recently published trial of a behavioral intervention to improve obstetrical care [28].

The analysis of the baseline data and the balanced randomization procedure will be performed by the study statistician, who will not be involved in the project management. Thus, there will be a clear separation between the generator of the intervention assignment and the study coordination [32].

### The intervention

#### Overview

Prenatal clinics allocated to the intervention group will receive an intervention divided in three phases and that will include the following activities; 1) Awareness phase: a) seminar to health providers and distribution of printed materials to increase awareness of the existence of the recommendations and program, b) identification of midwives interested in participating as facilitators of the program; 2) Training and persuasion phase: workshop for training on the “5 A’s” and for planning the implementation strategy; 3) Implementation phase: implementation of the program at the prenatal clinics identifying and training additional midwives to deliver the “5 A’s” and using reminders for health providers and patients. The intervention will be based on Roger’s “Diffusion of Innovations” theory [33] and will be tailored by formative research.

#### Theoretical basis

Roger’s “Diffusion of Innovations” theory [34] has been previously used in trials to implement programs at prenatal clinics, community schools, and home care associations [26]. Diffusion is defined as the process through which an innovation is communicated through channels over time among members of a social system [34]. An innovation is any idea, practice, service, or object that is considered new by an individual or social group, such as the “5 A’s” program. According to diffusion theory, certain characteristics of innovations increase the chances that they will be widely adopted. For example, the “5 A’s” program (innovation) would be more likely to be implemented and succeed if it is perceived as compatible with existing value systems and lifestyles. Roger’s theory describes five phases in the diffusion process: knowledge/awareness, persuasion, decision, implementation, and confirmation. The implementation of prenatal smoking cessation programs in prenatal clinics may follow a similar sequence with three phases: increase awareness about the importance and the availability of the programs; motivate, persuade, and train health providers; and planning and implementing the program.

We also based the intervention on two previously evaluated behavioral interventions based on Roger’s “Diffusion of Innovations” theory:

• The multifaceted intervention to promote the use of evidence-based childbirth practice evaluated by our group in Argentina and Uruguay, the Guidelines Trial [28]

• A behaviorally-based intervention designed to increase the number of hospitals that routinely provide effective smoking cessation programs in Queensland, Australia [26]

### Formative research

With the aim of ensuring that the intervention is effective, culturally-appropriate, and voluntarily integrated among women and health care providers into routine pregnancy care, formative research using qualitative and quantitative methods was carried out during the preparatory phase of the trial.

#### Qualitative component: focus groups and in-depth interviews

Focus groups and semi-structured interviews were conducted with prenatal care providers, hospital authorities and patients from selected public health care centers, which provide reproductive health and prenatal care in Buenos Aires, Argentina and Montevideo, Uruguay. The specific aims of this component were to identify the barriers and facilitators for the implementation of the “5 A’s” intervention at prenatal clinics, to assess pregnant smokers’ perspectives on adequate and readily accessible circumstances for the reception of the “5 A’s” strategy during routine prenatal care, and to assess pregnant women’s acceptability and opinions regarding specific self-help materials to increase sustainable smoking cessation during and after pregnancy.

#### Quantitative component: pilot study and preparatory survey

##### Pilot study

We will administer a questionnaire to women in the postpartum period admitted to six pre-selected maternity hospitals in Argentina (4 hospitals) and Uruguay (2 hospitals) where the outcomes will be measured. The objectives of the pilot study are to 1) pilot the questions to assess the main and secondary outcomes of the trial, 2) estimate the proportion of women who smoke and have received the “5 As” strategy during their prenatal care visits, among the women who deliver at the hospital, 3) assess agreement between saliva cotinine testing and self-reported smoking status, and 4) evaluate the proportion of women during postpartum hospital stay that consent to the questionnaire and saliva cotinine testing.

We will implement 200 interviews at each hospital during a one-month time frame (assuming refusals of slightly over 10%). Interviews will continue until we reach a total of 200 subjects at each hospital.

Trained interviewers, independent of the participating prenatal clinics, will be responsible for the administration of the questionnaire. Two shifts of interviews (one in the morning and one in the evening) will be organized to allow for interviewing women within the first 48 hours after delivery. The interviewers will identify all consecutive women that have undergone childbirth in the past 12 hours and that have received prenatal care at any of the participating prenatal clinics. After obtaining informed consent, they will proceed with the interview. This postpartum strategy for data collection has been proven feasible and successful in previous studies done by our group [35,36].

Each hospital must identify interviewers. Since the interview is relatively brief, some sites may prefer to have the health workers (e.g., nurses, nurse assistants) administer the survey interview. We will train the interviewers to screen, recruit, and interview the study participants.

Women will be informed about the study, invited to participate and asked to sign an informed consent. Women who agree to participate may refuse to answer any question and can withdraw from the study at any time. The informed consent will be read to the participant, her responses will be recorded, and a signed copy of the consent will be provided to the participant. Women may agree to answer the questionnaire and/or provide a saliva sample for cotinine test.

##### Preparatory survey

We will conduct this survey in all of the hospitals that will participate in the trial. The objectives of the study are: a) to estimate the number of women in a single month attending prenatal care at participating prenatal clinics who had their deliveries at the maternity hospitals; and b) to evaluate the process involved in a typical prenatal visit (e.g., average waiting time, personnel involved).

### Preparatory activities

To prepare the delivery of the intervention, the following activities will be conducted:

1. Tailoring the implementation intervention:

The results of the formative research will be used to refine the components of the intervention. Main points that will be confirmed or redefined will be: workshop format and length; recommended strategies to implement the smoking cessation program at the prenatal clinics; type and location of reminders; and educational materials to train health providers in the “5 A’s” for pregnant women and pregnancy-specific self-help materials to be provided to women within the “5 A’s” intervention.

2. *Adapting educational materials:*

a. Educational materials to train health providers in the 5 A’s.

The educational materials used in the workshops to train health providers conducted by the National Program of Tobacco Control in both countries do not include specific instructions on how to provide the “5 A’s” to pregnant women. We adapted pregnancy-specific materials from U.S. Agency for Healthcare Research and Quality (AHRQ) “Treating Tobacco Use and Dependence: Clinician's Packet. A How-To Guide For Implementing the Public Health Service Clinical Practice Guideline” [37] and from the UK National Institute for Health and Clinical Excellence’s guidelines for smoking cessation in pregnancy [38].

a. Pregnancy-specific self-help materials to be provided to women within the “5 A’s” intervention.

Currently, pregnancy-specific self-help materials are not provided to pregnant women by existing cessation programs. We will adapt materials for them from the same sources mentioned above for health providers.

3. Preparing seminars and workshops:

Contents of seminars and workshops will be defined, agendas will be detailed, and presentations and educational materials will be prepared and printed. The seminar and workshop will be piloted in Buenos Aires with a group of health providers working at similar but not participating prenatal clinics or maternity hospitals.

### Description of the intervention

The intervention will be tailored by formative research; therefore, the components described below could be modified after that stage.

#### Awareness phase

a) Seminar to health providers and distribution of printed materials

A seminar to all health professionals (physicians, nurses, and midwives) will be organized at the prenatal clinic in the hospital. The objectives of this component will be to present prenatal care providers with the problem of smoking during pregnancy, national recommendations for smoking cessation during pregnancy, the “5 A’s” intervention, the relevance of implementing the “5 A’s” intervention at the prenatal clinics, discuss activities planned with the implementation intervention.

b) Identification of facilitators

We intend to identify motivated midwives working at the prenatal clinics to serve as the facilitators who will be trained in the cessation program and then implement the program at their respective clinics. There are a number of reasons to select midwives over other types of health providers. In Argentina and Uruguay, midwives are trained to be primary prenatal caregivers and birth attendants. Midwives work as prenatal care providers at prenatal clinics in health centers. At hospitals, they mainly assist obstetricians. Thus, they have the opportunity and the necessary background to rapidly be trained in a smoking cessation program targeted to pregnant women. Women also respect the care and advice from midwives. Midwives have been proven as effective trained providers of the “5 A’s” in randomized-controlled trials [15]. Although physicians are prenatal care providers at hospitals, they usually dedicate a very short amount time to prenatal visits. In a study conducted in Mexico, Venezuela, and Panama in antenatal clinics, women spent a mean of 8–10 minutes in their physician’s office [39]. In another study performed in a public hospital in Buenos Aires among women receiving prenatal care, time spent with the provider was 14 minutes [40]. However, time spent at the clinic was longer (71–190 minutes in the four sites described); giving ample time for midwives to approach women for the implementation of the “5 A’s” intervention.

Nevertheless, if the formative research results in the consensus that general nurses and/or physicians can be included as facilitators, we will adapt this protocol to that purpose.

Facilitators will be identified by two ways after the initial seminar: 1) self nomination: those midwives that have an interest and are able to participate, and do not currently smoke, will be selected; 2) those midwives nominated by the health facilities’ authorities, and who are interested, will be selected. No initial limit in the number of facilitators will be considered. Considering the number of midwives available at the health facilities, we estimate that approximately 8–10 midwives will be selected per clinic.

#### Training and persuasion phase

Workshop for training on the “5 A’s” and for planning the implementation strategy.

The facilitators will participate in a 1.5 day workshop conducted in either Buenos Aires or Montevideo. Prenatal clinics’ health authorities will be invited to participate or to designate a delegate. The workshops will be given in previously selected sites outside of the prenatal clinics and will be conducted by a specialized trainer in smoking cessation programs. Interactive workshops have been proven an effective strategy for guidelines implementation [41].

The objectives of the workshop will be: to train facilitators on the “5 A’s” intervention (training of the trainers); to train facilitators on the suggested strategies to implement the “5 A’s” intervention program at their prenatal clinics; and to develop a dissemination and implementation plan.

The workshop will be adapted from the current workshops delivered by the National Tobacco Control Programs in Argentina and Uruguay [42]. Suggested topics to be included are: general overview of smoking prevalence and trend in Argentina/Uruguay; health consequences of smoking during pregnancy, specifically for the mother and the newborn baby; roles of the health care provider in preventing smoking among pregnant women; current recommendations and expected situation; brief explanation of smoking as an addiction; levels of interventions to promote smoking cessation; brief intervention “5 A’s”; assessing who is ready to quit and who is not; motivational interviews; role play; medications for smoking cessation; implementing the “5 A’s” in the prenatal clinic. We will also ask participants to create a plan to implement the “5 A’s.”

#### Implementation phase

During the implementation phase, each team of facilitators and prenatal clinic authorities will be responsible for organizing the “5 A’s” smoking cessation program in their prenatal clinics. The aim will be to ask all prenatal patients their smoking status and to then offer counseling and support included in the “5 A’s” intervention to all pregnant women who describe themselves as smokers. To this purpose, we will suggest to facilitators the following strategies to be considered in the implementation plan. First, they should identify the health providers who will deliver the “5 A’s” to pregnant women. Second, they will train the selected providers if they have not already received the training as facilitators. Finally, they will encourage use of reminders to be displayed in the prenatal clinic waiting rooms, clinical records, and the prenatal care.

### Activities in the control group

The control group will receive no intervention after randomization but will be asked to continue with standard in-service training activities. The control group will receive the intervention components after the intervention phase is completed.

### Activities in both intervention and control groups

The overview of the study will be presented to all participating prenatal clinics. The problem of smoking during pregnancy and the current national guidelines will be presented. We consider this presentation necessary to introduce the study and ethically justified to ensure that all providers have the same chance to know the importance of the problem and current national recommendations. In a previous clinical trial, this kind of activity was not likely to produce significant changes in providers’ behaviors [28].

### Measurement methods

#### Women’s outcome measurement

The frequency of women receiving the “5 A’s” at the end of pregnancy (primary outcome) and the frequency of women who smoke at the end of pregnancy (secondary outcome) will be measured in a survey conducted within the first 24 hours after delivery, during each woman’s hospital stay. Additionally, tobacco status among women who quit will be validated by cotinine analysis of saliva submitted within the first 12 hours postpartum.

##### Description of questionnaire to women

The survey will be conducted using a questionnaire in paper format that will include a set of core questions, including: basic demographic data (to be extracted from the clinical record); knowledge and attitudes regarding tobacco; tobacco use behaviors (specifically in the last two weeks before delivery); secondhand smoke exposure; and tobacco cessation counseling received during prenatal care. Questions on tobacco use and secondhand smoke exposure will be based on the previously validated questionnaire to assess tobacco use during pregnancy conducted in Argentina and Uruguay and other countries in 2005 [3,4]. Questions on tobacco cessation counseling received during prenatal care will cover the five steps of the “5 A’s” intervention: all women will be questioned as to whether they were asked their smoking status; smokers before pregnancy will be questioned as to whether they were advised to quit; women who smoked before pregnancy will also be asked if they were assisted in their attempt to quit by receiving skills and materials for tobacco cessation and if their counseling was monitored at every prenatal visit.

##### Questionnaire administration

The questionnaire was designed for face-to-face verbal administration by trained interviewers. Interviews will be conducted during the hospital stay after delivery. The questionnaire will take approximately 5–15 minutes to complete.

##### Cotinine analysis

We will measure cotinine levels in saliva during the immediate postpartum period, no later than 12 hours after delivery. A witnessed sample of saliva will be collected only in women that declared during the survey that they used to smoke but that they quit at some time during pregnancy. The sample will be collected by asking women to gently chew a Salivette sponge for 1–2 minutes until they feel it is saturated with saliva. The sponge will be put in a collection tube and stored for further analysis. This analysis will show if they were exposed to nicotine during the past several days. After the analysis is complete, the saliva sample will be thrown away according to biosafety procedures and will not be used for any purpose other than as previously stated. Samples will be kept in freezers and identified with the participant study number and shipped at the end of the collection period following all biosafety international requirements. Analysis of cotinine will be done by the Division of Laboratory Sciences of the Centers for Disease Control and Prevention in Atlanta, Georgia, USA, using gas chromatography and a pre-screening with an ELISA test.

Cotinine clearance has been reported to be higher in pregnancy, leading to a shorter half-life [43]. However, cotinine levels increase with gestational age and are more than twice as high at term than in the first and second trimesters [43].

### Attitude and readiness to change

We will assess the attitudes and readiness to provide counseling for smoking cessation to women (secondary outcome) following the “5 A’s” intervention in all prenatal care providers working at participating prenatal clinics. To that purpose, a self-administered questionnaire will be designed, adapted from a questionnaire developed and used by our group to evaluate birth attendants’ readiness to change in the Guidelines Trial [28].

#### Process measures

Process data will be collected during the intervention period at prenatal clinics included in the intervention group. The objectives of this process evaluation are to: 1) detect implementation problems that could be causal, in case the intervention was not effective; and 2) facilitate the replication of the intervention, in the event that it is proven effective. Twice during the intervention period (at the mid-way point and at the end of period), an external observer will monitor if the smoking cessation program is implemented and how it is organized.

#### Data collection and management

Outcome data will be collected in two six-month periods at all participating prenatal clusters: a baseline period and a follow-up period, separated by the intervention phase during which no outcome data will be collected. During the six-month data collection periods we will collect data on ≥200 women who have received prenatal care at each of the participating clinics. Outcome data will be collected in the maternity hospitals where women attending prenatal care at the participating prenatal clinics deliver. Process data will be collected during the intervention phase and only at intervention prenatal clinics.

Trained interviewers, independent of the participating prenatal clinics, will be responsible for the administration of the questionnaire to women, including obtaining the saliva samples. To allow for interviewing women within the first 12 hours after deliveries, two shifts of interviews will be organized: one in the morning and another in the evenings. The interviewers will identify all consecutive women who have undergone childbirth in the past 12 hours and who have received prenatal care at any of the participating prenatal clinics. After obtaining informed consent, they will interview each woman and obtain the saliva sample (if the woman is eligible). Demographic and childbirth data will be extracted from the clinical record. Cotinine samples will be kept refrigerated and then frozen in order to keep them in the appropriate conditions until shipment to CDC for analysis. This postpartum strategy for data collection has been proven feasible and successful in previous studies of our group [35,36].

Data will be collected on paper forms designed specifically for the study. A digital image of the interview form will be taken and sent to the local data center for data entry, but the inclusion form and the hard copy of the interview form will remain at the hospital until the end of the data collection period. With this system, it is ensured that personal identifiers will not be taken from the hospital and will be kept securely by the hospital staff.

The data forms will be entered in each country in a secure web data management system called OpenClinica, which is an open source software for clinical research studies using distributed data entry [44]. Digital pictures of the data forms will be sent encrypted to the data center by email. This system will allow for a digital backup of all study data forms, as well as a parallel second data entry of 10% of the forms to detect systematic errors at data entry. This method has been successfully used in other studies by our group.

### Statistical analysis

To test the primary hypothesis, we will consider the clusters of prenatal clinics as the unit of analysis. We are interested in observing how the absolute difference of the percentage of women who received the 5 A’s differs between the control and the intervention group. For that reason, we will compute the outcome rate for each prenatal clinic at baseline and the follow-up periods, and then we will calculate the outcome rate change as the difference between the follow-up and baseline rates. We will apply the *t*-test to those differences in order to test the intervention effect. It has been demonstrated that the *t*-test for two samples applied to cluster rates is appropriate when the number of clusters is low [45].

For the second hypothesis, we will use the woman as the unit of analysis because we want to study the effect of the intervention on the individual. For that we will fit a model in which the variables included will be the intervention, the time (baseline and follow-up measures) and the “intervention by time” interaction. To test the effect of the intervention we will focus on the significance of the interaction. We will use a Generalized Estimation Equation (GEE) to estimate the model and we will report the effect size as OR with the 95% confidence interval. For both outcomes, we will perform an intention-to-treat analysis to compare the groups.

To test if the intervention has changed the attitudes and readiness of midwives towards providing counseling to women, we will consider the percentage of providers that reported they are planning to change their approach about tobacco with their patients in the following six months or they have already changed it in the last six months. We will use the same analytical approach that will be used for the primary hypothesis.

### Sample size

The sample size is based on both primary and secondary objectives. A pre-test post-test (2 time–points) nested cross-sectional design was planned with 10 intervention and 10 control clinics. Each intervention and control group will contain seven clinics, with a minimum of 200 women at each time point, and three smaller clinics, with a minimum of 120 women at each time point. For each objective, the statistical power was estimated using a Monte Carlo simulation with 3,000 repetitions. In the simulation, binary outcomes from women were generated from the proposed pre-test post-test nested cross-sectional design under the following assumptions:

• An increase in the frequency of women receiving the “5 A’s” at the end of pregnancy from 10% to 20% in the control group and from 10% to 50% in the intervention group (i.e., an intervention effect of 30%) for the primary objective.

• A decrease in the frequency of women who smoke at the end of the pregnancy from 18% to 17% in the control group and from 18% to 12% in the intervention group (i.e., an intervention effect of 5%) for the secondary objective.

• An intra-cluster correlation between 2 outcomes from different women at different times (pre-test/post-test) = Intra-cluster correlation between 2 different women at the same time of 0.05. The value of the latter intra-cluster correlation coefficient was observed in a study carried out in Argentina and Uruguay [3].

For this sample size, the estimated statistical power at the 5% level of significance (2-sided) is 1.0 for the primary objective and 0.89 for the secondary objective.

### Limitations

The cotinine analysis will allow us to prevent differential non-disclosure of tobacco status between women who declared that they quit smoking at some time during pregnancy in the intervention and control clinics, which can bias the effect estimation on the smoking prevalence [46]. However, it is possible that by assessing cotinine after delivery, we may not detect some women smokers who had not smoked in the last 3–4 days because of labor, delivery, and hospital admission, who also did not self-report smoking. These potential false negative results may pose a limitation in correctly estimating the real prevalence of smokers across all clinics, but will not alter the estimation of the effect of the intervention strategy.

### Ethical aspects

The protocol and the informed consent documents were submitted and approved by: the Ethics Committees of all participating hospitals; the Ethics Committee of the Ministry of Health of the Province of Buenos Aires, Argentina; the Ethics Committee of the Centro de Educación Médica e Investigaciones Clínicas “Norberto Quirno” (CEMIC); Ethics Committee of the Scholl of Medicine, Universidad de la República, Uruguay, and Tulane University IRB.

## Competing interests

The authors declare that they have no have competing interests.

## Authors’ contributions

PB, JB, and FA had the original idea and designed the intervention and the first protocol. FA, PB, JB, AM, MB, AB, LG and PM wrote the manuscript, in collaboration with MC, AA, AC, LL, MGBG, VTT, SLF, RAS, PM. Dietz, CJ, PB. All authors have given final approval of the manuscript.
